# Book Notices

**Published:** 1880-02-20

**Authors:** 


### *BOOK NOTICES.*

A MANUAL OF THE PRACTICE OF SURGERY, by W. FAIRLIE CLARKE, M.A. and M.B. F. R. S., Assistant Surgeon to Charming Cross Hospital. From the last London edition, revised and edited, with additions, by an American Surgeon. 299 pp. Illustrated. New York: William Wood & Co. 1879.

This is an excellent work, containing as nearly, perhaps, as possible in a small and compact volume, the recent advances in surgical knowledge. There are valuable suggestions and additions by the American editor. It is certainly very practical and desirable to the surgeon and practitioner.

THE VENERIAL DISEASES, including Stricture of the Male Urethra, by E. L. KEYES, A M., M.D., Professor of Dermatology and Adjunct Professor of Surgery in the Bellevue Hospital; Consulting Surgeon to the Charity Hospital, etc., etc. New York: Wm. Wood & Co., 27 Great Jones street. 1880.

This is an illustrated work of 242 pages, octavo in cloth illustrated, and beautifully gotten out, and constitutes another issue of the series of standard medical authors, by William Wood & Co. It is a most instructive and valuable work and should be in the library of every practitioner.

MALIGNANT DEGENERATION OF A FIBROID TUMOR OF THE UTERUS—LARGE FALSE ANEURISM IN THE SUBSTANCE OF THE GROWTH. DRS. ALBERT N. BLODUETT AND CLIFTON E. WING. Boston.

RETROVERSION IN RELATION TO LACERATIONS OF THE CERVIX UTERI, Etc., by NATHAN BOZEMAN, M.D. New York. Reprint from volume III. Gynecological Transactions. 1879.

